# Health care service for families with children at early risk of developmental delay: an All Our Families cohort study

**DOI:** 10.1111/dmcn.14343

**Published:** 2019-08-30

**Authors:** Matthew J Russell, Shainur Premji, Sheila Mcdonald, Jennifer D Zwicker, Suzanne Tough

**Affiliations:** ^1^ Community Health Sciences, Cumming School of Medicine University of Calgary Calgary AB Canada; ^2^ School of Public Policy University of Calgary Calgary AB Canada; ^3^ PolicyWise for Children & Families University of Calgary Calgary AB Canada; ^4^ Paediatrics, Cumming School of Medicine University of Calgary Calgary AB Canada; ^5^ Department of Kinesiology University of Calgary Calgary AB Canada

## Abstract

**Aim:**

This study examined children’s health care service use, mothers’ workforce participation, and mothers’ community engagement based on children’s risk of developmental delay.

**Method:**

We used data from the All Our Families study, a prospective pregnancy cohort. Ages and Stages Questionnaire (ASQ) scores at year 2 indicated risk of developmental delay. To investigate the impact of risk of developmental delay when children were not diagnosed, a sensitivity analysis excluded reports of neurodevelopmental disorder (NDD) diagnosis at year 3. Outcomes were maternal reports of children’s health and allied health visits (and estimated costs), and maternal workforce participation and community engagement from year 2 to 3.

**Results:**

Among 1314 mother–child dyads, 209 (16%) children were classified as being at risk of developmental delay by the ASQ, and 42 (3%) had a reported diagnosis of NDD. Risk of developmental delay was related to increased use of allied health care services (incidence risk ratio 5.04 [year 3]; 95% confidence interval 2.49–10.2) and health visits (incidence risk ratio 1.33 [year 3]; 95% confidence interval 1.14–1.54). The average expected allied health costs were greater for children at risk versus not at risk of developmental delay. However, when excluding children with reported diagnoses of an NDD from this analysis, increased service use and costs in the remaining at‐risk population were not observed. Community engagement and workplace participation among families did not differ on the basis of risk of developmental delay.

**Interpretation:**

These results suggest increased health care service use by families of children at risk of developmental delay is driven by those receiving a diagnosis of an NDD in the subsequent year.

**What this paper adds:**

- Early developmental delay risk was related to health care service use and costs.
- Diagnosis of neurodevelopmental disorder drove increased health care service use and costs.
- Early developmental delay risk did not relate to parental workforce participation.
- Early developmental delay risk did not relate to community engagement participation.

## What this paper adds

- Early developmental delay risk was related to health care service use and costs.
- Diagnosis of neurodevelopmental disorder drove increased health care service use and costs.
- Early developmental delay risk did not relate to parental workforce participation.
- Early developmental delay risk did not relate to community engagement participation.

This article's abstract has been translated into Spanish and Portuguese.

Follow the links from the http://onlinelibrary.wiley.com/doi/10.1111/dmcn.14343/abstract to view the translations.

## Resumen

**Objetivo:**

Este estudio examinó el uso del servicio de atención médica de los niños en riesgo de retraso en el desarrollo, y a su vez la participación de las madres en el mercado laboral y su participación comunitaria basada en el riesgo de los niños de presentar retraso en el desarrollo.

**Método:**

Utilizamos datos del estudio All Our Families, una cohorte de embarazo longitudinal. Las puntuaciones del Cuestionario de edades y etapas (ASQ) a los 2 años se usaron para identificar riesgo de retraso en el desarrollo. Para investigar el impacto del riesgo de retraso del desarrollo cuando los niños no fueron diagnosticados, un análisis de sensibilidad excluyó los informes de diagnóstico de trastornos del neurodesarrollo (NDD) a los 3 años. Los resultados se recolectaron de informes maternos de salud infantil y visitas de salud a diferentes profesionales de la salud (y costos estimados), y la participación materna en el mercado laboral y la participación materna en la comunidad entre el 2 a 3 año de vida de sus hijos.

**Resultados:**

Entre 1.314 díadas madre‐hijo, 209 (16%) niños fueron clasificados como en riesgo de retraso en el neurodesarrollo por el ASQ, y 42 (3%) tenían un diagnóstico informado de NDD. El riesgo de retraso en el desarrollo se relacionó con un mayor uso de servicios de atención médica por diversos profesionales de la salud (incidencia cociente de riesgos 5.04 [año 3]; Intervalo de confianza del 95% 2.49–10.2) y visitas de salud (riesgo de incidencia relación 1,33 [año 3]; 95% intervalo de confianza 1.14–1.54). La proyección de los costos promedio de salud aliada esperada fueron mayores para los niños en riesgo versus no en riesgo de retraso en el desarrollo. Sin embargo, al excluir a los niños con diagnósticos informados de un NDD de este análisis, no se observaron un aumento en el uso del servicio y los costos en la población en riesgo restante. Cuando se comparó la participación en la comunidad y la participación en el mercado laboral entre las familias, los resultados no difirieron en función del riesgo de retraso en el desarrollo de los niños.

**Interpretación:**

Estos resultados sugieren que el mayor uso de servicios de atención médica por parte de las familias de los niños en riesgo de retraso del desarrollo son impulsados por aquellos que reciben un diagnóstico de NDD en el año siguiente.

## Abstract (pt)

**Objetivo:**

Este estudo examinou o uso de serviços de saúde por crianças, participação das mães na força de trabalho, e engajamento das mães na comunidade com base no risco da criança para atraso no desenvolvimento.

**Método:**

Usamos dados do estudo All Our Families, uma coorte prospectiva de gestantes. Os escores no Questionário Idades e Fases (QIF) na idade de 2 anos indicou risco de atraso no desenvolvimento. Para investigar o impacto do risco para atraso no desenvolviemnto quando crianças não tinham diagnóstico, uma análise de sensibilidade excluiu os relatos de desordem neurodesenvolvimental (DND) na idade de 3 anos. Os desfechos foram os relatos maternos sobre a saúde da criança e visitas a serviços de saúde (com custos estimados), e a participação das mães na força de trabalho e engajamento na comunidade nos anos 2 e 3.

**Resultados:**

Entre 1314 díades mãe‐criança, 209 (16%) crianças foram classificadas como tendo risco para atraso no desenvolvimento, e 42 (3%) tiveram diagnóstico de DND. O risco de atraso no desenvolvimento foi relacionado a um aumento no uso de serviços aliados de saúde (razão do risco de incidência 5,04 [ano 3]; intervalo de confiança a 95% 2,49–10,2) e consultas de saúde (razão do risco de incidência 1,33 [ano 3]; intervalo de confiança a 95% 1,14–1,54). A média esperada dos custos dos serviços aliados de saúde foi maior para crianças de risco versus aquelas sem risco para atraso no desenvolvimento. No entanto, quando excluídas da análise as crianças com diagnóstico reportado de DND, o maior uso dos serviços e custos no restante da população de risco não foram observados. O engajamento na comunidade e participação na força de trabalho entre famílias não diferiu com base no risco para atraso do desenvolvimento.

**Interpretação:**

Os resultados sugerem que o uso aumentado de serviços de saúde por famílias com crianças com risco para atraso no desenvolvimento é causado por aqueles que receberam diagnóstico de DND no ano subsequente.

**Abbreviations**
ASQ: Ages and Stages Questionnaire
NDD: Neurodevelopmental disorder

Neurodevelopmental disorders (NDDs) are a heterogeneous group of conditions (e.g. autism, intellectual disability, speech delays, and cerebral palsy) with onset in the first 5 years of life, characterized by impairments in motor, cognitive, and social functioning.^1^, ^2^, ^3^ The prevalence of NDDs is estimated to be from 5% to 9% of children.^4^ Prevalence depends on age, definition of NDD, and data source.^4^ Among Canadian children with a disability, over 75% also have an NDD,^5^ and children with NDDs often experience activity limitations throughout their lifespan that affect their quality of life.^5^, ^6^ Children with NDDs have higher health care service use (three times more hospitalization and two times more physician visits than those without NDDs),^4^, ^7^ are more likely to be among the top 5% of the costliest health care users (e.g. 51% of children with cerebral palsy are high cost users),^8^ are more prone to mental health problems (e.g. 85% of persons with autism have a mental health disorder over their life),^9^, ^10^ and are more likely to make use of government disability support programmes than children without NDDs.^8^ Access to services for persons with NDDs is a priority for Canadians, as ratified in the United Nations Convention on the Rights of Persons with Disabilities, and a key priority for children with NDDs and for their families.^7^, ^11^

Identification of NDDs and access to support provide the foundation to address lifelong needs of children with NDDs.^12^ In particular, early childhood intervention for children with NDDs is thought to improve developmental outcomes across the lifespan more than later intervention.^12^, ^13^ This has led to the creation of early intervention programmes to improve outcomes for children with NDDs.^14^ However, one key aspect of these programmes is that they often require a formal diagnosis of an NDD, which creates a critical barrier to the use of potential early support services.^12^ Receiving early access to support services is important to families as it can provide a foundation to addressing lifelong challenges associated with children’s participation in society.^15^

Tools have been developed to increase early identification of children at risk of developmental delay, which often correspond to a later diagnosis of an NDD.^6^ For example, the Ages and Stages Questionnaire (ASQ) was developed to help identify children at risk of developmental delay in research and clinical practice.^6^ The ASQ has five domains (communication, gross motor, fine motor, problem solving, and personal social abilities), which are normed with children at a similar age and provide information on potential developmental delays. This approach has some alignment with the International Classification of Functioning, Disability and Health approach, targeting specific functional domains that may limit a child’s ability to participate in society (https://www.who.int/classifications/icf/en/). The ASQ has also been shown to have reasonable sensitivity and specificity as a tool for identifying NDDs, with risk predicting later diagnosis of NDD.^6^

The primary responsibility for the access and coordination of support services for children at risk of developmental delay or with a diagnosis of an NDD lies largely with families.^5^ This affects the family; over a child’s lifespan, families report an estimated average annual out‐of‐pocket cost of Can$10 000 to Can$30 000,^7^ reduced working hours and labour force participation,^7^ and a loss of community social supports.^16^ Caregivers also report a negative psychological toll; they contend with higher levels of stress, feelings of isolation and frustration, and physical and mental health issues than families without children with NDDs.^17^, ^18^ Unfortunately, we know little about the impact of risk of developmental delay and diagnoses of NDDs on families during the early years and the supports they may be receiving.

In this study we used self‐reported data from a prospective pregnancy cohort to assess how risk of developmental delay through the ASQ for children at the age of 2 years related to allied health and health care service visits for the children, and community engagement and workforce participation for mothers reported at age 3 years, based on their previous year. A sensitivity analysis was also used to assess the role of diagnosis of an NDD in child and parent outcomes when the child was 3 years old.

## Method

This study was approved by the Conjoint Faculties Research Ethics Board at the University of Calgary (reference REB 15‐3027). All participants gave written informed consent to the research and the publication of the results.

### The All Our Families pregnancy cohort

We used data from the All Our Families study (previously All Our Babies), a prospective pregnancy cohort based in Calgary, Alberta, Canada, on approximately 3000 medically low‐risk mothers and their children.^19^, ^20^, ^21^ The women were medically low‐risk because they were recruited at community‐based medical services at the beginning of their pregnancy. Participants were not recruited from high‐risk obstetric practices and tertiary medical clinics. Detailed descriptions of this cohort are available elsewhere.^19^, ^20^, ^21^ Briefly, this cohort was recruited during pregnancy by a community based multi‐method strategy using community settings, primary health care offices, and community laboratory services. This strategy resulted in a diverse sample of women who were representative of the sociodemographic of the population in the Greater Calgary area. Initial recruitment began in 2008 and was completed in 2010. Participants were eligible if they spoke English, were between 6 days and less than 24 weeks’ gestation at enrolment, were at least 18 years old, and lived in the Greater Calgary area and planned to stay there during pregnancy. Mothers were asked to complete questionnaires when their child was 2 and 3 years old, as well as at other time points (https://allourfamiliesstudy.com).

#### Participant follow‐up

Participants who agreed to follow‐up were contacted and invited to participate.^19^, ^20^, ^21^ The sample size was less than the initial total in subsequent data collection points because of funding, attrition, and eligibility due to questionnaire timing constraints. To encourage continuing participation, the study team contacted participants by telephone and email if questionnaire data were missing, required clarification, or if participants did not return a questionnaire.

#### Attrition

Of the 3200 original mothers in the cohort, 2106 were eligible for year 2 data collection, with 1596 completing questionnaires (75.8%), and 2909 mothers were eligible for year 3 data collection, with 1994 completing these questionnaires (69%). As our target analyses required an intersection between the two data collection points, we focused on mothers who completed both year 2 and year 3 questionnaires (1314 mothers). Mothers who continued in the study were more likely to be older, partnered, have higher educational attainment, have higher income, and to have been born in Canada.^21^

### Exposure: risk of developmental delay and diagnosis of NDDs

Our exposure was the ASQ (Third Edition) scores at year 2.^6^ The ASQ has five domains: communication, gross motor, fine motor, problem solving, and personal social abilities. We defined exposure by ‘ASQ risk’ as showing monitor (−1 SD) scores on any two of the five domains of the ASQ, a definition used in recent research shown to follow expected developmental delay proportions.^22^ We also created a second exposure group (‘ASQ risk no NDD’), to remove service use related to diagnosis and treatment of reported NDD by excluding diagnoses of NDDs reported at year 3. Diagnoses of NDDs included mothers’ reports of developmental delays, cerebral palsy, autism, epilepsy, and attention‐deficit/hyperactivity disorder.

### Outcomes: service use, workforce participation, and community engagement

Our outcomes were mothers’ reports of child health care visits, workforce participation, and community engagement in year 3, based on their previous year. These outcomes were based on previously reported outcomes for families caring for children with NDDs.^7^, ^16^ We calculated mothers’ reports of the total number of health visits as the sum of family doctor, paediatrician, and developmental paediatrician visits; and allied health visits as the sum of psychologist, social worker, occupational therapy, speech therapy, physiotherapy, and dietician visits. Outcome variables were also generated on the basis of cost estimates for these two types of service. Published average wages for each type of visit were derived using the Alberta Schedule of Medical Benefits and the Alberta Salary and Wage Survey, 2017 (physician wages: Schedule of Medical Benefits, 1st April 2017; other wages: Alberta Learning and Information Services, 29th May 2018). Costs were inflated to 2018 Canadian dollars using the Bank of Canada inflation calculator (accessed 29th May 2018). We used conservative cost estimates, assuming that each visit lasted 1 hour and was a general consultation (see Appendix S1, online supporting information for cost details). For workforce participation, we investigated mothers’ reports of work hours per week, categorized as full‐time (>30h/wk) or part‐time (≤30h/wk). Finally, for community engagement, we investigated whether mothers reported any use (yes) or no use (no) of the library, recreational facilities, parenting groups, drop‐in parenting groups, informal play groups, drop‐in child care, or other group activities.

### Independent variables

For our multivariable analyses, we adjusted for maternal age at delivery, educational status, income, marital status, ethnicity, parity, workforce participation, and social support (coding described in Table 1 and above). Social support was a standardized measure based on the National Longitudinal Survey of Children and Youth Social Support Scale. Independent variables were chosen on the basis of their potential to confound relationships with maternal and child outcomes.^22^

**Table 1:** Maternal participants’ characteristics for the full sample and split by Ages and Stages Questionnaire (ASQ) risk status for developmental delay

Participants’ characteristics	Full sample (*n*=1314)	Not at risk (*n*=1062)	At risk (*n*=209)
Mean maternal age (SD), y:mo	31:5 (4:4)	31:5 (4:4)	31:6 (4:5)
Marital status	1306	1055	209
Married/common law	1252 (95.9)	1010 (95.7)	204 (97.6)
Single/separated/divorced/widowed	54 (4.1)	45 (4.3)	5 (2.4)
Education	1305	1055	208
High school or less	100 (7.7)	78 (7.4)	17 (8.2)
Beyond high school	1205 (92.3)	977 (92.6)	191 (91.8)
Annual household income (before taxes)	1254	1012	200
<Can$60 000	158 (12.6)	123 (12.2)	30 (15.0)
≥Can$60 000	1096 (87.4)	889 (87.8)	170 (85.0)
Parity (number of children to reach live birth)	1298	1049	207
One	644 (49.6)	508 (48.4)	116 (56.0)^a^
More than one	654 (50.4)	541 (51.6)	91 (44.0)^a^
Ethnicity	1305	1055	208
White	1086 (83.2)	897 (85.0)	159 (76.4)^a^
Other	219 (16.8)	158 (15.0)	49 (23.6)^a^

Data are *n* (%) unless otherwise stated. Age differences were compared using a two‐sample *t*‐test and proportions were compared using *χ* ^2^ tests of association.

a Reflects significant association for participant characteristic and ASQ risk status, *p*<0.05.

### Data analysis

STATA version 15 (StataCorp, College Station, TX, USA) was used for all statistical analyses. Descriptive statistics included means, standard deviations, medians, interquartile ranges, frequencies, and proportions. Pearson’s *χ* ^2^ tests, *t*‐tests, and Fisher’s exact test (when expected cell counts were fewer than 5) were used to compare demographic factors between exposure groups.

Next, analyses were used to describe how ASQ risk status related to children’s and mothers’ outcomes. After tests of normality (using Shapiro–Wilks tests) for the visit and costing data, we used non‐parametric equality of medians tests to compare the median number of health and allied health visits between groups, and Mann–Whitney *U* tests to compare children’s average health and allied health visit costs. Pearson’s *χ* ^2^ tests were used to describe how ASQ risk status related to mothers’ workforce participation and community engagement.

Finally, we used multivariable regression models to estimate the relation between ASQ risk status and children’s health visits and costs. Independent variables included maternal age at delivery, education, income, marital status, ethnicity, parity, social support, and workforce participation. For health and allied health use, we assessed for evidence of over‐dispersion (using the alpha test) and zero‐inflation (using the Vuong test). Both models exhibited over‐dispersion while neither model demonstrated evidence for zero‐inflation. We therefore estimated the expected incidence risk ratios (the ratio between expected visits for ASQ risk status and not‐at‐risk status from year 2 to year 3) using negative binomial regression models. For health and allied health costs, we first log‐transformed our cost data, then used a linear regression model to estimate log‐costs, and finally back‐transformed the data for estimates of mean cost differences between risk groups. We used log‐transformation and linear regression models as these are common approaches to estimating average costs. All models used a manual backwards stepwise approach to determine the final, adjusted estimates. Significance was determined using *p*<0.05. Heteroscedasticity was evaluated within all linear regression models, and we used the linktest command in Stata to evaluate goodness‐of‐fit for the negative binomial and linear regression models respectively.

## Results

### Maternal participant characteristics

The mean age of mothers at childbirth was 31 years 5 months (SD 4y 5mo). Ninety‐six per cent of mothers were married or in a common‐law relationship, 92% had at least some postsecondary education, 87% had a household income of at least Can$60 000, 50% had only one live birth (for parity), and 83% were white (Table 1). Maternal participant characteristics were mostly statistically equivalent between ASQ at‐risk and not‐at‐risk groups, except that mothers with children at risk of developmental delay were more likely to have one live birth (vs more than one) and to be another ethnicity (vs white) than mothers with not‐at‐risk children.

### Child at risk of developmental delay and diagnosis of NDDs

Overall, 96.7% of our sample provided a response to the ASQ questions at age 2 years. Comparison of the sample characteristics between those who responded and those who did not indicated that those who did not respond were more likely to be of ethnicity other than white (*p*=0.04). The ASQ (age 2y) showed 14% of children at risk of communication delay, 13% of gross motor delay, 11% of fine motor delay, 14% of problem‐solving delay, and 15% of personal social delay (Table 2). Moreover, 16% of children showed risk of delay in at least two domains of the ASQ at year 2 (our ‘at‐risk’ group). This definition aligns with percentages found in the literature related to developmental delay of 12% to 16%.^22^ At year 3, 3% of all mothers reported a diagnosis of NDDs. A much larger percentage of ASQ at‐risk children had a diagnosis of an NDD (12.9%; 27 out of 187) than not‐at‐risk children (1.4%; 15 out of 1047).

**Table 2:** Child Ages and Stages Questionnaire (ASQ) risk at year 2 for each domain, number of domains at risk (including and excluding diagnosis of neurodevelopmental disorder [NDD]), and number of diagnoses of NDDs at year 3

ASQ	*n* (%)
Communication (*n*=1285)	
Not at risk	1110 (86.4)
At risk (monitor score) −1 SD	175 (13.6)
Gross motor (*n*=1288)	
Not at risk	1115 (86.6)
At risk (monitor score) −1 SD	173 (13.4)
Fine motor (*n*=1283)	
Not at risk	1144 (89.2)
At risk (monitor score) −1 SD	139 (10.8)
Problem solving (*n*=1281)	
Not at risk	1104 (86.2)
At risk (monitor score) −1 SD	177 (13.8)
Personal social (*n*=1286)	
Not at risk	1097 (85.3)
At risk (monitor score) −1 SD	189 (14.7)
Overall ASQ domains at risk (*n*=1271)	
0–1 domains at risk (not at risk)	1062 (83.6)
2–5 domains at risk (at risk)	209 (16.4)
0–1 domains at risk, no diagnosis of NDD	1046 (85.2)
2–5 domains at risk, no diagnosis of NDD	182 (14.8)
Diagnosed with NDD at 36mo (*n*=1271)	
Yes	42 (3.3)
No	1229 (96.7)

### Health care service use and costs

**Figure 1:**
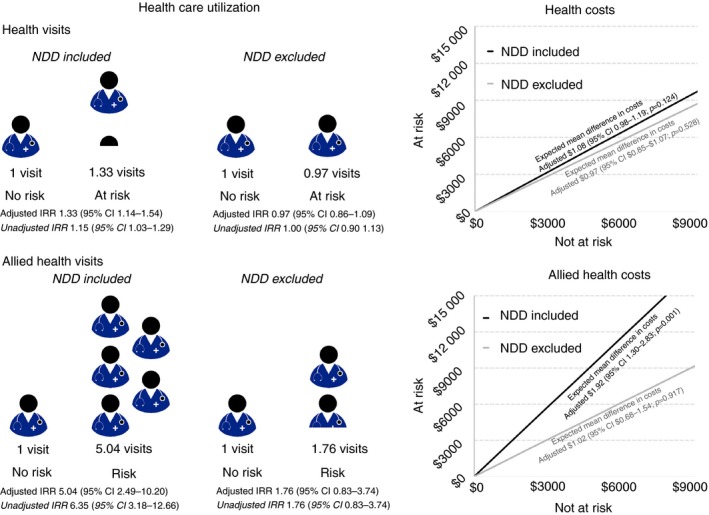
Health care use for health visits and allied health visits at year 3 (over the past year). Expected health and allied health visits are reported to the left as an incidence risk ratio (IRR; year 3) and expected health and allied health care costs (the ratio of at‐risk to not‐at‐risk costs) are reported to the right. Cost ratios are shown including and excluding diagnoses of a neurodevelopmental disorder (NDD)

Children at risk of developmental delay used more health and allied health care services than children who were not at risk (Table 3). This use was increased for allied health care services, compared with health care services, and both remained when adjusting for the independent variables. In line with these increases, we found that cost increases (seen by incidence risk ratios) were only seen for allied health visits, not health visits. This increase remained for visits when controlling for our independent variables (incidence risk ratio [year 3]: 5.04) and amounted to an estimated average of Can$1.92 in allied health spending for at‐risk children for every Can$1 spent on children not at risk of developmental delay (Fig. 1). A sensitivity analysis removing children identified with an NDD found that differences in use and costs were driven by children with identified diagnoses at year 3.

**Table 3:** Health visits (crude), costs (crude), workforce participation, and community engagement reported at year 3 on the basis of Ages and Stages Questionnaire (ASQ) risk status, and calculated including and excluding diagnoses of a neurodevelopmental disorder (NDD)

Resource	Not at risk (*n*=1062)	At risk (*n*=209)	*p*
Health visits (past year)	Median (IQR)	Median (IQR)	
Health visits (number)	2 (2)	3 (2)	0.052
Allied health visits (number)	0 (0)	0 (1)	<0.001
Health visit costs (past year)	Mean (SD, 95% CI)	Mean (SD, 95% CI)	
Health visit cost (Can$)	241.96 (243.47, 227.30–256.62)	279.36 (389.04, 226.30–332.41)	0.050
Allied health visit cost (Can$)	29.77 (201.11, 17.66–41.88)	185.67 (767.69, 80.98–290.36)	<0.001
Workforce participation	*n* (%)	*n* (%)	
Part‐time (<30h/wk)	675 (63.6)	136 (65.1)	0.677
Full‐time (≥30h/wk)	387 (36.4)	73 (34.9)	
Community engagement	*n* (%)	*n* (%)	
Yes	1044 (98.3)	206 (98.6)	0.788
No	18 (1.7)	3 (1.4)	

	Not at risk, no NDD (*n*=1047)	At risk, no NDD (*n*=182)	*p*
Health visits (past year)	Median (IQR)	Median (IQR)	
Health visits (number)	2 (2)	2 (2)	0.262
Allied health visits (number)	0 (0)	0 (0)	0.027
Health visit costs (past year)	Mean (SD, 95% CI)	Mean (SD, 95% CI)	
Health visit cost (Can$)	235.61 (205.77, 223.13–248.09)	237.01 (173.96, 211.57–262.45)	0.442
Allied health visit cost (Can$)	18.29 (83.61, 13.21–23.35)	32.23 (125.36, 13.89–50.56)	0.027
Workforce participation	*n* (%)	*n* (%)	
Part‐time (<30h/wk)	664 (63.4)	121 (66.5)	0.427
Full‐time (≥30h/wk)	383 (36.6)	s61 (33.5)	
Community engagement	*n* (%)	*n* (%)	
Yes	1030 (98.4)	179 (98.4)	0.981
No	17 (1.6)	3 (1.6)	

Adjusted estimates of health visits and costs are shown in Figure 1. IQR, interquartile range; CI, confidence interval.

### Workforce participation and community engagement

Analyses of workforce participation and overall community engagement revealed no effect of ASQ risk status for either. A sensitivity analysis comparing percentages of mothers with no employment, part‐time employment, and full‐time employment by ASQ risk status also did not find a difference. Removing confirmed NDDs from the analysis did not change this finding. However, community engagement analyses split by type of resource use showed one difference by ASQ risk status. Mothers with children at risk of developmental delay were less likely to use informal play groups than mothers with children who were not at risk (at risk of developmental delay: 112 of 209 [53.59%]; not‐at‐risk: 672 of 1062 [63.28%]; *p*=0.008). This relationship remained, but had a higher *p* value, when we excluded diagnoses of an NDD (at risk by ASQ use: 100 of 182 [54.95%]; not‐at‐risk: 664 of 1047 [63.42%]; *p*=0.03).

## Discussion

Using a community sample of mothers from Calgary, Alberta, we identified 16% of children at risk of developmental delay through the ASQ at year 2, and 3% of children with a diagnosis of an NDD at year 3. These NDD rates are lower than prevalence rates among older children reported in Canada (6–10y; 8.3% in British Columbia),^4^ and replicate previous findings suggesting lower identification of NDDs in the early years.^23^

For health care service use, we found a higher number of allied health and health visits for children identified at risk of developmental delay, compared with children who were not at risk. Similarly, we found corresponding allied health cost differences that were greater for children at risk compared with those not at risk. This has implications for caregivers as some allied health visit costs may need to be covered by caregivers directly and/or require them to go through processes to apply for funding.^24^ The differences in health care use and costs were largely accounted for by children who received a diagnosis of an NDD by year 3. This suggests that increases in health care use were driven by children who received a diagnosis of an NDD.

Increases in health care visits may involve visits related to diagnosis, as well as services provided to address increased NDD‐related needs. Typically, diagnosis requires a substantial series of testing,^2^ which involves access to resource‐intensive clinical assessments among physicians and other health care providers. As a result, it would be expected that any child with a recent diagnosis of an NDD would have more visits with health care providers as a result of the process. Beyond visits for diagnosis, some early health care‐based services for children with an NDD and their families may be unavailable in the absence of a diagnosis, as well as other support programmes (e.g. education or disability supports).^24^, ^25^ As some early supports are connected to diagnosis, one risk for families is that they may have unidentified children who could benefit from early support. It also begs an important question, as identification is connected to the use of services: how can we ensure individuals in need receive support? For example, our research suggests that the use of identification tools such as the ASQ may help discover some children with an NDD. However, how to best use such tools is still in contention. For example, although a 2016 Canadian task force recommended against population‐level implementations of screening of children without clear symptoms on the basis of weak evidence,^26^ the Canadian Paediatric Society recommended an enhanced 18‐month well‐child visit using screening tools as a way to begin discussions on child development.^27^ Furthermore, the World Health Organization stresses the importance of creating appropriate environments to support screening programmes, with agreed standards on who to treat, knowledge of the cost and benefits of treatment, and continued follow‐up plans.^28^, ^29^ Future discussion is needed on how best to identify children with early developmental support needs. This discussion is particularly important as early identification with tools such as the ASQ and intervention could benefit children by improving their developmental outcomes such that they potentially avert a later diagnosis of an NDD.

Finally, despite children scoring at risk of developmental delay on one of the ASQ domains, we generally saw little difference in maternal workforce participation and community engagement. This suggests there may be larger lifestyle changes for families in the later years, once children have a confirmed diagnosis of an NDD, with research showing decreased workforce participation for caregivers^7^ and decreased community engagement in later years.^16^ Future research is necessary to elucidate why this difference is only noticeable in later years. Despite this finding, we must note that it does not reflect the emotional well‐being of the parent, who may be experiencing stress associated with adapting to demands of parenting a child at risk of developmental delay. Further research is needed to better understand the early psychological impact of children at risk of developmental delay on families.

### Limitations

This study had several limitations. First, as the ASQ measure is based on the mother’s perception of her child, this leaves open the possibility of misclassification bias. Despite this possibility, we should note that previous research suggests that the ASQ with parental report has fair properties for use as an identification tool for NDDs.^6^ Second, health care use and cost estimates may differ from actual numbers. In particular, cost estimates are probably lower than actual numbers, because of our conservative costing definitions. Our costing estimates exclude additional testing and services that may be offered to this population for diagnosis and treatment, some of which have been noted in recent studies on NDDs.^4^ As such, future research should use more detailed administrative data to obtain more exact estimates of health care use and costs. Third, we should note that the coding of use of community supports may lack the sensitivity necessary to detect subtle changes in support use. As such, future research might use more sensitive questions to address these potential changes, such as Likert scales or qualitative inquiry. Last, the demographics of this sample broadly represented the parenting population in a large urban city with access to a universal health care system, with the exception of retention of families with slightly higher incomes. This may somewhat limit the generalizability. For example, family income has been noted to affect health care use patterns, suggesting potential differences if we used a less affluent target population.^30^ Similarly, the exclusion of those unable to complete the questionnaire in English, and the differing characteristics of mothers who continued the study and answered the ASQ may limit generalizability.

## Conclusion

This study suggests that the majority of children at risk of developmental delay identified through the ASQ did not have more visits with physicians or allied health professionals from the age of 2 to 3 years, relative to children who were not at risk. Instead, the increases in health care visits and costs were primarily driven by children who ultimately received a diagnosis of an NDD at age 3 years. This finding supports the importance of creating strategies to identify children with NDD‐related needs in the early years. We found that 3% of children were identified before age 3 years with an NDD, which is lower than estimated rates of 6% to 9% in middle childhood.^4^, ^23^ This finding may indicate that some children with an NDD are not benefiting from allied health care services in the preschool years. Further research is needed about how to best provide services and support to families of children at risk of developmental delay before the age of 3 years.

## Supporting information


**Appendix S1** **: **Outcome costs in Alberta, Canada.
