# Supplementary material for: Health care service for families with children at early risk of developmental delay: an All Our Families cohort study
Source: Dev Med Child Neurol. 2019 Aug 30;62(3):338–45. doi: 10.1111/dmcn.14343 (PMC7028137; doi:10.1111/dmcn.14343)
Supplement: Supplementary file 1 — Appendix S1 : Outcome costs in Alberta, Canada. [file DMCN-62-338-s001.doc]

**Data supplement**

**1.0 Outcome costs** *(in Alberta, Canada)*

*Health visits*

1. Family doctor or developmental pediatrician ($82.43)

*Allied health visits*

1. Psychologist ($49.13)
2. Social worker ($21.25)
3. Occupational therapist ($43.93)
4. Speech therapist ($50.19)
5. Physiotherapist ($45.26)
6. Dietician ($43.41)
